# The Sphingosine Kinase 2 Inhibitor ABC294640 Restores the Sensitivity of BRAFV600E Mutant Colon Cancer Cells to Vemurafenib by Reducing AKT-Mediated Expression of Nucleophosmin and Translationally-Controlled Tumour Protein

**DOI:** 10.3390/ijms221910767

**Published:** 2021-10-05

**Authors:** Petra Grbčić, Thomas O. Eichmann, Sandra Kraljević Pavelić, Mirela Sedić

**Affiliations:** 1 Department of Biotechnology, University of Rijeka, Radmile Matejčić 2, 51000 Rijeka, Croatia; petra.grbcic@biotech.uniri.hr; 2 Institute of Molecular Biosciences, University of Graz, Heinrichstraße 31/III, 8010 Graz, Austria; thomas.eichmann@uni-graz.at; 3 Faculty of Health Studies, University of Rijeka, Viktora Cara Emina 5, 51000 Rijeka, Croatia; sandrakp@uniri.hr; 4 Centre for Applied Bioanthropology, Institute for Anthropological Research, Ljudevita Gaja 32, 10000 Zagreb, Croatia

**Keywords:** colon cancer, BRAFV600E, PLX4032, vemurafenib, sphingolipids, sphingosine-1-phosphate, sphingosine kinase 2, ABC294640, opaganib, nucleophosmin, translationally-controlled tumour protein

## Abstract

Vemurafenib (PLX4032), small-molecule inhibitor of mutated BRAFV600E protein, has emerged as a potent anti-cancer agent against metastatic melanoma harboring BRAFV600E mutation. Unfortunately, the effect of PLX4032 in the treatment of metastatic BRAF mutated colorectal cancer (CRC) is less potent due to high incidence of fast-developing chemoresistance. It has been demonstrated that sphingolipids are important mediators of chemoresistance to various therapies in colon cancer. In this study, we will explore the role of major regulators of sphingolipid metabolism and signaling in the development of resistance to vemurafenib in BRAF mutant colon cancer cells. The obtained data revealed significantly increased expression levels of activated sphingosine kinases (SphK1 and SphK2) in resistant cells concomitant with increased abundance of sphingosine-1-phosphate (S1P) and its precursor sphingosine, which was accompanied by increased expression levels of the enzymes regulating the ceramide salvage pathway, namely ceramide synthases 2 and 6 and acid ceramidase, especially after the exposure to vemurafenib. Pharmacological inhibition of SphK1/SphK2 activities or modulation of ceramide metabolism by exogenous C6-ceramide enhanced the anti-proliferative effect of PLX4032 in resistant RKO cells in a synergistic manner. It is important to note that the inhibition of SphK2 by ABC294640 proved effective at restoring the sensitivity of resistant cells to vemurafenib at the largest number of combinations of sub-toxic drug concentrations with minimal cytotoxicity. Furthermore, the obtained findings revealed that enhanced anti-proliferative, anti-migratory, anti-clonogenic and pro-apoptotic effects of a combination treatment with ABC294640 and PLX4032 relative to either drug alone were accompanied by the inhibition of S1P-regulated AKT activity and concomitant abrogation of AKT-mediated cellular levels of nucleophosmin and translationally-controlled tumour protein. Collectively, our study suggests the possibility of using the combination of ABC294640 and PLX4032 as a novel therapeutic approach to combat vemurafenib resistance in BRAF mutant colon cancer, which warrants additional preclinical validation studies.

## 1. Introduction

Vemurafenib (PLX4032), a small-molecule inhibitor of mutated BRAFV600E protein, has emerged as a potent anti-cancer agent against metastatic melanoma harbouring BRAFV600E mutation that occurs in codon 600 resulting in the substitution of a valine for a glutamic acid (V600E) leading to a constitutive activation of the BRAF protein. The latter promotes cell proliferation in the absence of necessary growth factors under normal conditions. However, the effect of vemurafenib in the treatment of metastatic BRAF mutated colorectal cancer is less potent than in BRAFV600E melanoma, and its clinical efficacy as monotherapy in BRAF mutant metastatic colorectal cancer is reduced due to the high incidence of fast-developing chemoresistance. Previously, it has been shown that BRAF mutant colon cancer cell lines have higher levels of phospho-protein kinase B (AKT) indicative of an activation of the phosphoinositide 3-kinase (PI3K)–AKT pathway when compared to BRAF mutant melanoma [1]. Importantly, BRAF mutant colon cancer cell line HT-29, with acquired resistance to vemurafenib, displayed an increased expression of activated AKT. As expected, pharmacological inhibitors of the phosphoinositide 3-kinase (PI3K)–AKT pathway in combination with vemurafenib showed improved outcomes both in vitro and in vivo [1,2]. Apart from AKT signalling, other oncogenic signalling pathways, including the epidermal growth factor receptor, also play important roles in the development of resistance to vemurafenib in colorectal cancer. Specifically, BRAF inhibition suppresses phosphorylated forms of the extracellular signal-regulated kinase (ERK) resulting in a down-regulation of its negative feedback circuit and activation of the epidermal growth factor receptor pathway, which activates the RAS (Rat sarcoma) protein and the mitogen-activated protein kinase kinase/mitogen-activated protein kinase (MEK/MAPK) signalling axis via a direct activation of CRAF or by promoting the formation of BRAF–CRAF heterodimers [3]. However, many gaps remain in our current understanding of the acquired resistance to BRAF inhibition in BRAF mutant colorectal cancer that preclude successful management of the colorectal cancer patients carrying BRAF mutation.

Sphingolipids have been described as important mediators of chemoresistance in various therapies when treating colon cancer. Therefore, it is no surprise that the enzymes regulating sphingolipid turnover represent potential drug targets. Preponderant experimental evidence has demonstrated that pharmacological inhibition of sphingosine kinase isoforms (either sphingosine kinase 1 or 2) renders resistant colon cancer cells more responsive to both standard and targeted therapies. For example, the administration of sphingosine kinase inhibitor SKI increased oxaliplatin cytotoxicity and apoptosis induction in the BRAF mutant RKO colon cancer cell line [4]. Furthermore, pharmacological inhibition of sphingosine kinase 1 (SphK1) by *N,N*-dimethyl-sphingosine (DMS) or SphK1 silencing in cetuximab-resistant colon cancer cells increased their response to cetuximab [5]. ABC294640 (3-(4-chlorophenyl)-N-(4-pyridinylmethyl)-tricyclo (3.3.1.13,7) decane-1-carboxamide), a first-in-class sphingosine kinase 2 (SphK2) inhibitor undergoing clinical trials, has shown promising results in reversing chemoresistance in colon cancer. Specifically, co-treatment of HT-29 cells with a low concentration of ABC294640 markedly increased the sensitivity of HT-29 cells to 5-fluorouracil or cisplatin and facilitated apoptosis induction triggered by this chemotherapy [6]. Moreover, oral administration of ABC294640 supressed HT-29 xenografts growth in nude mice, which puts forward that targeting sphingosine kinase 2 by ABC294640 could provide a novel therapeutic opportunity for BRAF mutant colon cancer.

These findings led us to explore the role of sphingolipid metabolism in the development of resistance to vemurafenib in BRAF mutant colon cancer cells and to investigate the possibility of targeting sphingolipid metabolic regulators to counteract vemurafenib resistance. We found significantly increased expression levels of activated sphingosine kinases 1 and 2 in resistant cells concomitant with increased abundance of sphingosine-1-phosphate (S1P) and its precursor sphingosine, which was accompanied by a marked increase in the expression levels of the enzymes regulating the ceramide salvage pathway, including ceramide synthases 2 and 6 and acid ceramidase, especially after the exposure to vemurafenib. As expected, the pharmacological inhibition of sphingosine kinase 1/sphingosine kinase 2 activities or modulation of ceramide metabolism by exogenous C6-ceramide markedly enhanced the anti-proliferative effect of vemurafenib in resistant RKO cells in a synergistic manner. It is important to note that the pharmacological inhibition of sphingosine kinase 2 by ABC294640 proved effective at restoring the sensitivity of resistant cells to vemurafenib in the largest number of combinations of sub-toxic drug concentrations with minimal cytotoxicity compared to either sphingosine kinase 1 inhibitor or C6 ceramide. For this reason, the combination of ABC294640 and vemurafenib was selected for further mechanistic study. We found that enhanced anti-proliferative, anti-migratory, anti-clonogenic and pro-apoptotic effects of the combination treatment with ABC294640 and vemurafenib relative to either drug alone coincide with the inhibition of S1P-regulated AKT activity and abrogation of AKT-mediated cellular levels of nucleophosmin and translationally-controlled tumour protein. Collectively, our study suggests the possibility of using the combination of ABC294640 and vemurafenib as a novel therapeutic approach to combat vemurafenib resistance in BRAF mutant colon cancer. This possibility warrants further studies in additional preclinical models.

## 2. Results

### 2.1. Development and Molecular Characterisation of Vemurafenib-Resistant RKO Colon Cancer Cell Line Harbouring BRAFV600E Mutation

Vemurafenib (PLX4032)—resistant colon cancer cell line carrying BRAFV600E mutation (RKOr) was developed by exposing the parental RKO cell line to successively increasing concentrations of PLX4032 throughout the period of 6 months until a clinically relevant concentration of 11.52 µM [7] was reached. The development of resistance was monitored by measuring the IC_50_ values of vemurafenib in parental and resistant cells by the MTT assay, which showed a 10-fold increase in the IC_50_ in resistant vs. the parental RKO cell line (Supplementary Table S1). PLX4032-resistant cells had distinct morphological features characterized by a spindle-like shape, increased intercellular separation and the formation of pseudopodia (Supplementary Figure S1) associated with epithelial-to-mesenchymal transition characteristic for resistant phenotype of colorectal cancer cells [8]. Consistent with previous findings in literature [1,9], we confirmed elevated expression levels of phospho-c-RAF, phospho-ERK1/2, phospho-MEK1/2 and phospho-AKT in resistant cell lines under basal conditions and especially upon the treatment with PLX4032 (Supplementary Figure S2), which again demonstrated that activation of the RAF/MEK/ERK and AKT signalling could be associated with the development of vemurafenib-resistant phenotype in colon cancer.

### 2.2. Altered Regulation of Sphingosine-1-Phosphate and Ceramide Metabolism Underpins Acquired Resistance to Vemurafenib in BRAF Mutated Colon Cancer Cells

**Figure 1:**
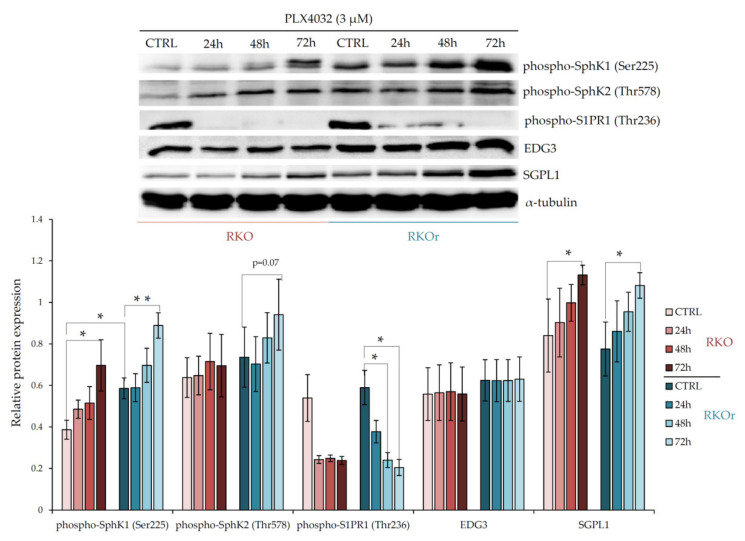
Time-dependent changes in relative expression of the enzymes regulating S1P metabolism and signalling in sensitive (RKO) and resistant (RKOr) cells treated with PLX4032 (3 µM). Relative protein expression was measured using Quantity One software for densitometry analysis of western blot bands. Data represent mean and standard deviation obtained from three independent biological experiments. α-tubulin was used as loading control. Statistical significance is denoted with an asterisk (* *p* < 0.05, ** *p* < 0.01). SphK1 (Sphingosine kinase 1), SphK2 (Sphingosine kinase 2), S1PR1 (EDG1, sphingosine-1-phosphate receptor 1), EDG3 (S1PR3, sphingosine-1-phosphate receptor 3), SGPL1 (sphingosine-1-phosphate lyase 1).

Prompted by the previous findings demonstrating the role of the enzymes regulating sphingosine-1-phosphate (S1P) and ceramide metabolism as mediators of chemoresistance in colon cancer [10,11,12] and based on an earlier observation from the study in BRAFV600E mutated melanoma which revealed altered ceramide/S1P ratio in vemurafenib-resistant cells [13], we sought to investigate the possible involvement of the key regulators of S1P and ceramide turnover and signalling in acquired resistance to vemurafenib in BRAF mutated colon cancer cells. For this purpose, parental RKO and resistant RKOr cells were cultured either with or without 3 µM vemurafenib corresponding to the IC_50_ concentration in sensitive cell line for 24, 48 and 72 h, and the expression levels of the key metabolic and signalling regulators of bioactive sphingolipids were measured by western blot. First, we examined the differences between parental and resistant cell lines in the levels of sphingosine kinases 1 (SphK1) and 2 (SphK2) that catalyse the phosphorylation of sphingosine to produce pro-survival S1P. At the basal level (in the absence of PLX4032), the expression of phospho-SphK1 was significantly increased (*p* < 0.05) in resistant cells than in sensitive cells, whilst phospho-SphK2 expression showed a modest increase in resistant cells (Figure 1). However, the expression levels of both phospho-SphK1 and phospho-SphK2 were markedly elevated in a time-dependent manner in resistant cells relative to sensitive ones after the exposure to vemurafenib peaking at 72 h (Figure 1), which indicates PLX4032-induced activation of SphK1 and SphK2 in resistant cells. Interestingly, the expression level of sphingosine-1-phosphate lyase 1 (SGPL1), an enzyme important for S1P degradation, was not remarkably different between parental and resistant cell lines grown without or with PLX4032.

Next, we investigated the expression of S1P receptors S1PR1 and EDG3 (S1PR3) that transduce S1P signalling within the cells. The basal levels of both, phospho-S1PR1 and EDG3 were slightly up-regulated in resistant vs. parental cells, albeit this difference was not statistically significant (Figure 1). Similarly, modest albeit non-significant increase in the EDG3 expression levels were observed in resistant cells exposed to PLX4032 in comparison with sensitive cells. Strikingly, an inverse effect was observed with phospho-S1PR1, whose expression decreased in a time-dependent manner in both cell lines. These results indicate that the S1PR1- and S1PR3-mediated signalling is not the major determinant of non-responsiveness to vemurafenib in colon cancer cells.

**Figure 2:**
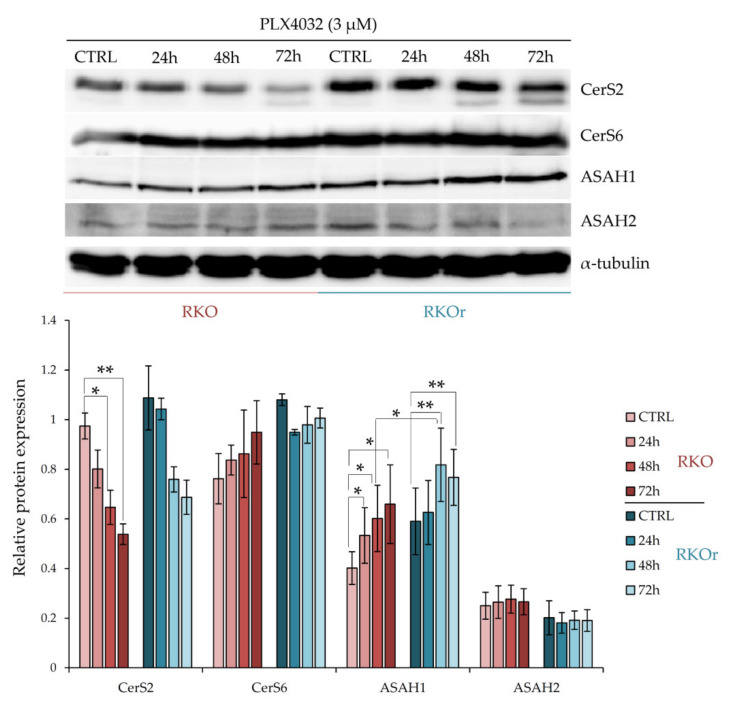
Time-dependent changes in relative expression of the enzymes regulating the ceramide salvage pathway in sensitive (RKO) and resistant (RKOr) cells treated with PLX4032 (3 µM). Relative protein expression was measured using Quantity One software for densitometry analysis of western blot bands. The data represent mean and standard deviation obtained from three independent biological experiments. α-tubulin was used as loading control. Statistical significance is denoted with an asterisk (* *p* < 0.05, ** *p* < 0.01). CerS2 (ceramide synthase 2), CerS6 (ceramide synthase 6), ASAH1 (acid ceramidase), ASAH2 (neutral ceramidase).

We next questioned whether the mechanisms of acquired resistance to vemurafenib involved differential regulation of ceramide metabolism, specifically ceramide synthesis from sphingosine catalysed by ceramide synthases 2 and 6, and ceramide degradation into sphingosine and free fatty acids catalysed by acid (ASAH1) and neutral (ASAH2) ceramidases. Ceramide synthases (CerS) catalyse synthesis of ceramides with different acyl chain lengths. CerS2 (LASS2) is responsible for the synthesis of ceramides containing mainly C20, C22, C24:0, C24:1 and C26 fatty acids, whereas CerS6 (LASS6) mainly generates ceramides with C14:0, C16:0 and C18:0 acyl chains [14]. The western blot analysis showed increased baseline levels of CerS2 and CerS6 in resistant cells (Figure 2). Exposure to PLX4032 progressively elevated the levels of CerS6 in a time-dependent manner in both cell lines although with a greater extent observed in resistant cells. On the other hand, the treatment with PLX4032 reduced the levels of CerS2 in both cell lines; but the resistant cells challenged with PLX4032 still expressed higher levels of CerS2 across all three time points, although without statistical significance.

The baseline levels of ASAH1 were modestly increased in resistant cells when compared to their sensitive counterparts (Figure 2). Importantly, the incubation with vemurafenib progressively increased ASAH1 levels in both cell lines in a time-dependent manner and significantly higher levels of ASAH1 were detected after a 48-hourtreatment in resistant cells relative to sensitive cells, and this trend persisted after a 72-h treatment with vemurafenib. At the same time, there were no marked differences in ASAH2 levels between sensitive and resistant cells cultured with or without vemurafenib.

To further investigate the role of the sphingomyelinase pathway leading to sphingomyelin hydrolysis into ceramide in the development of resistance to vemurafenib, we measured the expression of acid sphingomyelinase (ASM) and neutral sphingomyelinase 1 (NSmase1) in parental and resistant cells grown with or without vemurafenib. The expression levels of ASM and NSmase1 were not significantly different between sensitive and resistant cells regardless of the treatment conditions (Supplementary Figure S3).

Collectively, the obtained results demonstrated an increased expression of activated SphK1 and SphK2 in resistant vs. sensitive cells, especially after the exposure to vemurafenib. In addition, resistant cells expressed higher levels of ceramide-producing enzymes CerS2 and CerS6, as well as ceramide-degrading enzyme ASAH1, whose expression was potentiated in resistant cells after the treatment with vemurafenib. Thus, impairment in the regulation of S1P production accompanied by metabolic imbalance in ceramide salvage pathway could play a role in increased resistance to cytostatic effects of vemurafenib in BRAF mutant colon cancer cells.

### 2.3. Mass Spectrometry-Based Sphingolipidomic Analysis Confirms Dysregulation of S1P Metabolism as the Mechanism Underlying Vemurafenib Resistance in BRAF Mutant Colon Cancer Cells

**Figure 3:**
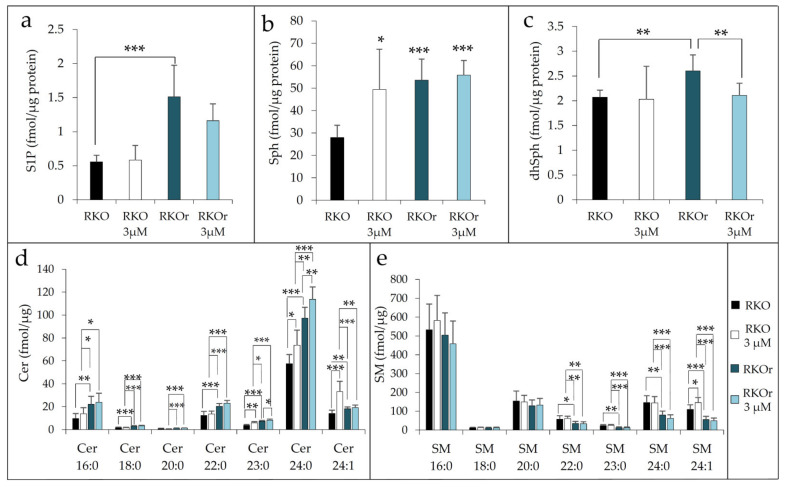
Sphingolipidomic analysis of different sphingolipid species using ultra-high-performance liquid chromatography coupled with triple quadrupole mass spectrometry (UHPLC/MS/MS) in sensitive (RKO) and vemurafenib-resistant (RKOr) cells under basal conditions (no treatment) and treated with 3 µM PLX4032 for 48 h. Cellular lipids were extracted, quantified by mass spectrometry and normalized to protein content. Results are expressed as fmol/µg protein concentration and represent the levels of S1P (**a**), sphingosine (Sph) (**b**), dihydrosphingosine (dhSph) (**c**), ceramides (Cer) (**d**) and sphingomyelins (SM) (**e**). Asterisk denotes statistical significance (* *p* < 0.05, ** *p* < 0.01, *** *p* < 0.001). Results were obtained from three individual biological experiments performed in two technical replicates.

To further confirm the findings of western blot analysis indicating the aberrant regulation of metabolism of bioactive sphingolipids, we measured the abundance of S1P, sphingosine (Sph) and dihydrosphingosine (sphinganine, dhSph) along with several ceramide and sphingomyelin (SM) species in sensitive and resistant cells cultured in the absence or presence of 3 µM vemurafenib for 48 h using ultra-high-performance liquid chromatography coupled with triple quadrupole mass spectrometry (UHPLC/MS/MS). We detected significantly increased basal levels of S1P in resistant vs. sensitive cells, and this pattern of S1P production was sustained in resistant cells after the treatment with vemurafenib (Figure 3a). Increased abundance of S1P in resistant cells results from enhanced activity of SphK1 and SphK2 previously revealed by western blot analysis (Figure 1). The baseline levels of S1P precursor sphingosine were also significantly elevated in resistant when compared to sensitive cells, and this difference reached statistical significance under the treatment conditions with vemurafenib (Figure 3b). The increased abundance of sphingosine in resistant cells was likely to result from significant up-regulation of ASAH1 expression, which was potentiated by vemurafenib exposure (Figure 2). It was noted that parental cells exerted marked upregulation of Sph level after the exposure to vemurafenib. Given that free sphingosine is cytotoxic, its elevation in sensitive cells exposed to vemurafenib is likely to be associated with the induction of stress response mechanisms as a part of cellular response to vemurafenib in these cells, rather than with steering the metabolism towards the production of pro-survival S1P metabolite, which appears to be the case with resistant cells.

Previous data (Figure 2) led us to hypothesize that CerS2/CerS6-catalysed ceramide formation through the salvage pathway is augmented in resistant cells in comparison to their sensitive counterparts. Further confirmation was provided by sphingolipidomic analysis which revealed a statistically significant increased abundance of long-chain ceramide 16:0 and very long-chain ceramides 22:0/24:0 produced by CerS6 and CerS2, respectively, in resistant cells when compared to sensitive cells under basal conditions, and their levels further increased in resistant cells after the vemurafenib challenge (Figure 3d). The same pattern was observed with ceramide 18:0, 20:0 and 23:0, albeit the concentration of this saturated species was generally much lower. Furthermore, the basal level of dihydrosphingosine (sphinganine) (dhSph), precursor in de novo ceramide synthesis, was significantly increased in resistant cells (Figure 3c), indicating that the mechanisms underlying acquired resistance to vemurafenib could possibly include modulation of different routes of ceramide synthesis. Strikingly, the concentration of dhSph in resistant cells markedly dropped to the levels detected in sensitive cells after the treatment with vemurafenib (Figure 3c), suggesting that vemurafenib either supported de novo synthesis of ceramides or due to increased SphK activities previously observed (Figure 1), that dhSph served as a substrate for the formation of dhS1P, a S1P analogue with similar bioactive properties. It was also interesting to note that vemurafenib treatment gave rise to a statistically significant decline in the levels of several sphingomyelin species including 22:0, 24:0 and 24:1 in resistant cells (Figure 3e), which paralleled increased abundance of their corresponding constituent ceramide species (Figure 3d).

To sum up, the findings from sphingolipidomic analyses lend further support to increased production of S1P and its precursor Sph in resistant cells under both basal and treatment conditions, which was accompanied by elevated levels of specific long-chain and very long-chain ceramides and reduced abundance of their cognate sphingomyelin species after vemurafenib exposure.

### 2.4. Pharmacological Manipulation of S1P/Ceramide Metabolism Restores the Sensitivity of Resistant BRAF Mutant Colon Cancer Cells to PLX4032 in a Synergistic Manner

Prompted by the findings that vemurafenib tilts metabolic balance towards the production of S1P and (very) long-chain ceramide species in resistant cells, we sought to further investigate whether pharmacological inhibition of S1P-producing enzymes SphK1 and SphK2 or reprograming of the ceramide metabolism by adding exogenous short-chain C6-ceramide could reverse resistance to vemurafenib. In order to achieve this, RKOr cells were treated with increasing concentrations of PLX4032 (1.5, 3, 7.5, 15 and 30 µM, IC_50_ value, Supplementary Table S1) together with either C6-ceramide, PF543 (a selective inhibitor of SphK1) or Opaganib (Yeliva^®^, ABC294640), a first-in-class selective SphK2 inhibitor. For all three latter compounds, three different concentrations were tested—starting from the corresponding IC_50_ concentration (Supplementary Table S2) down to two-fold consecutively decreased sub-IC_50_ concentrations (Table 1).

The obtained results showed that either the pharmacological inhibition of SphK1/SphK2 activities or the modulation of ceramide metabolism by exogenous C6-ceramide markedly enhanced the anti-proliferative effect of PLX4032 in resistant RKO cells in a concentration-dependent manner (Table 1) when compared to a single PLX4032 treatment (Supplementary Table S1). Importantly, all three agents were able to augment the anti-tumour activity of vemurafenib even at low, sub-toxic concentrations. Although C6-ceramide had the most pronounced stimulatory effect on the anti-tumour activity of vemurafenib, this combination also exerted the most potent cytotoxic effect (LC_50_ < 5 µM). Oppositely, co-treatment with ABC294640 and PLX4032 elicited lowest cytotoxicity, which renders this drug combination interesting in terms of potential clinical application.

To determine the nature of the response for each tested drug combination in resistant RKO cells, we calculated the combination index (CI) values using CompuSyn software, where CI < 1, =1, and >1 indicated synergism, additive effect and antagonism, respectively. The combination of vemurafenib with C6-ceramide, PF-543 or ABC294640 exhibited synergistic anti-proliferative effects (Table 2), especially when each of the two drugs in combination was used at relatively low, sub-toxic concentrations (sub-IC_50_ values). Of these, only two combinations of PLX4032 with each, either C6-ceramide or PF-543, had synergistic effects when sub-IC_50_ values of each compound were combined (Table 2). Importantly, co-treatment with ABC294640 and PLX4032 resulted in a synergistic, cytostatic effect observed with six drug combinations where sub-IC_50_ values of each drug were used. Given that the major aim of synergistic drug combinations is to reduce the dose of a drug in order to decrease toxicity without compromising therapeutic efficacy, our findings suggest that ABC294640 and PLX4032 represent a promising drug combination for vemurafenib-resistant cells that works synergistically over a wide range of low sub-IC_50_ concentration combinations.

**Figure 4:**
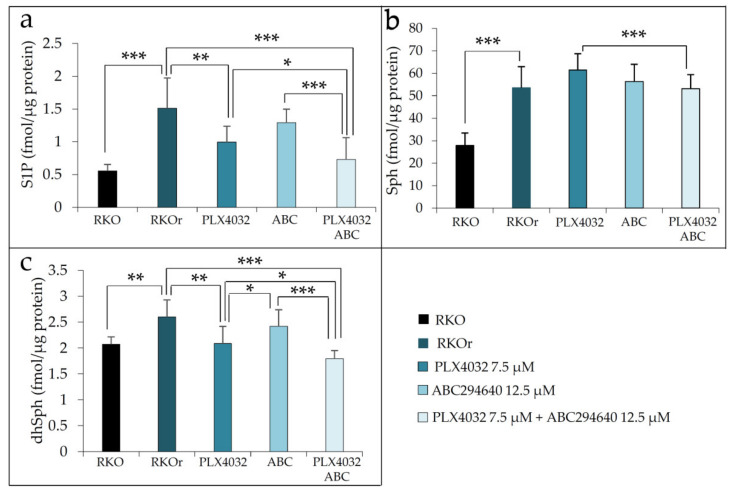
Sphingolipidomic analysis of different sphingolipids species using ultra high-performance liquid chromatography coupled with triple quadrupole mass spectrometry (UHPLC/MS/MS). Cells were cultured in the presence or absence of the drugs for 48 h. Results are expressed as fmol/µg protein concentration and represent the levels of S1P (**a**), sphingosine (Sph) (**b**) and dihydrosphingosine (dhSph) (**c**). Asterisks mark statistical significance (* *p* < 0.05, ** *p* < 0.01, *** *p* < 0.001). Results were obtained from three individual biological experiments performed in two technical replicates. ABC (ABC294640), PLX (PLX4032).

To additionally confirm the efficacy of combining ABC294640 and PLX4032 in reducing vemurafenib resistance in BRAF mutant colon cancer, we established the second BRAF mutated colon cancer cell line HT-29r with acquired resistance to vemurafenib, as confirmed by a 6.7-fold higher IC_50_ value of vemurafenib in this cell line vs. parental HT-29 cells (Supplementary Table S1). When HT-29r cells were treated with IC_50_ and two sub-IC_50_ concentrations of ABC294640 (Supplementary Table S2) together with vemurafenib, anti-proliferative effects of vemurafenib were remarkably potentiated (Supplementary Table S3) when compared to single vemurafenib treatment (Supplementary Table S1). Importantly, the co-administration of ABC294640 and vemurafenib produced synergistic, cytostatic activity in HT-29r with seven concentration combinations where sub-IC_50_ concentrations of each drug were employed (Supplementary Table S4). Based on the obtained results, we hypothesized that enhanced anti-proliferative activity of combination treatment with vemurafenib and SphK2 inhibitor ABC294640 in comparison to vemurafenib alone could be attributed to down-regulation of S1P production. Indeed, sphingolipidomic analyses revealed a statistically significant decrease in S1P level in resistant RKOr cells exposed to combination treatment with vemurafenib and ABC294640 at their lowest synergy-producing sub-IC_50_ concentrations (7.5 and 12.5 µM, respectively) when compared to either PLX4032 or ABC294640 alone (Figure 4a).

Interestingly, the levels of S1P precursor sphingosine (Sph) were slightly increased after both single-agent treatments with PLX4032 and ABC294640 when compared to untreated RKOr cells, and combined treatment was able to modestly reduce Sph to a level measured under basal conditions (Figure 4b). Furthermore, single-agent PLX4032 induced significant decrease in dhSph levels in resistant cells in comparison to untreated RKOr cells. It is important to note that more pronounced reduction in dhSph level was achieved with the combined treatment when compared to an individual single drug treatment (Figure 4c).

Altogether, the obtained data point to the relevance of S1P metabolism in the development of resistance to vemurafenib and indicate that targeting S1P production by the SphK2 inhibitor ABC294640 could increase the sensitivity of BRAF mutated resistant colon cancer cells to vemurafenib.

### 2.5. Inhibition of SphK2 Activity by ABC294640 Augments PLX4032-Induced Apoptosis and Anti-Migratory and Anti-Clonogenic Effects in Resistant RKOr Cells

**Figure 5:**
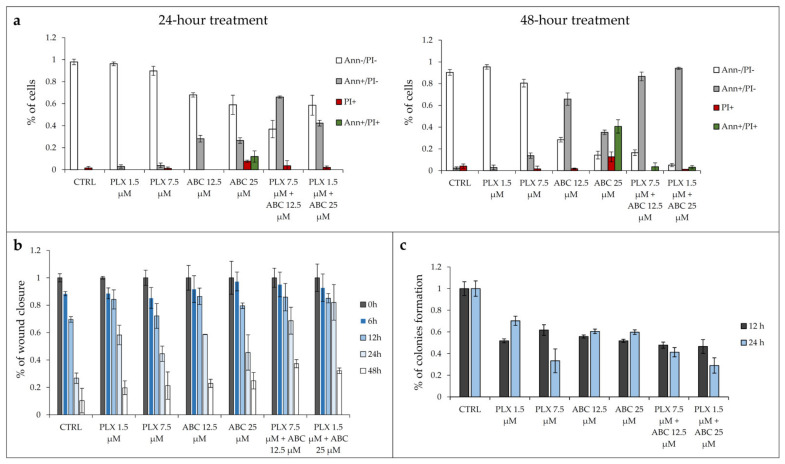
Effect of combination treatment with ABC294640 and PLX4032 on apoptosis induction, migration and colony forming ability of vemurafenib-resistant RKOr cell line. (**a**) Annexin V assay was performed to detect induction of apoptosis when cells were treated with either single agents or their combination for 24 and 48 h. Data were obtained from two independent biological experiments in two technical replicates and represent the percentage of viable cells (Ann-/PI-), early apoptotic cells (Ann+/PI-), late apoptotic/primary necrotic cells (Ann+/PI+) and secondary necrotic cells (PI+). Ann (Annexin V); PI (propidium iodide). (**b**) A wound healing assay was performed in RKOr cells treated with either single agents or their combination for 6, 12, 24 and 48 h. Data were obtained from two individual biological experiments performed in technical duplicates. (**c**) A clonogenic assay was caried out in RKOr cells treated with either single agents or their combination for 12 and 24 h followed by a one-week incubation to allow the formation of colonies. Data were obtained from two individual biological experiments performed in technical duplicates. PLX (PLX4032), ABC (ABC294640).

In an additional attempt to better understand why concomitant exposure to vemurafenib and ABC294640 produced a more potent anti-cancer effect in resistant RKOr cells in comparison with either drug alone, individual drugs or their combinations were assayed for the effects on the induction of apoptosis and inhibition of cell migration and clonogenic capacity. For this purpose, the two lowest sub-IC_50_ concentrations of each drug were selected based on their ability to produce synergistic effects in resistant cells. The potential of combined therapy to induce apoptosis was evaluated by the Annexin V assay (Figure 5a). RKOr cells were treated with either two sub-IC_50_ concentrations of PLX4032 alone (1.5 and 7.5 μM), ABC294640 (12.5 and 25 μM) or their combinations for 24 and 48 h. As expected, single-agent PLX4032 did not have a profound inhibitory effect on cell viability in resistant RKOr cells in both tested concentrations after 24-h treatment (Figure 5a). In contrast, single treatment with ABC294640 induced early apoptosis by 28.21% at a 12.5 μM concentration, whereas 25 μM triggered early apoptosis by 26.50% accompanied by increased occurrence of late apoptotic and necrotic cells by 11.96% and 7.70%, respectively (Figure 5a).

Similarly, the 48-h treatment with vemurafenib at 1.5 µM did not exert remarkable inhibitory effect on the cell viability, whereas higher concentrations had only modest pro-apoptotic effect mirrored by slightly increased proportion of early apoptotic and necrotic cells by 13.78% and 1.6%, respectively (Figure 5a). The pro-apoptotic effect of single-agent ABC294640 at 12.5 μM observed after a 24-h treatment was strongly potentiated after 48 h, as demonstrated by a 2.3-fold rise in the proportion of early apoptotic cells. The same trend, albeit with a higher magnitude, was detected with 25 µM ABC294640 after 48 h, as evidenced by a marked reduction in the viable cell population and an upsurge in the percentage of cells that entered late apoptosis/primary necrosis and secondary necrosis by 40.63% and 12.70%, respectively.

Although individual treatments with PLX4032 failed to produce any meaningful pro-apoptotic effect in resistant cells regardless of the treatment conditions, its combination with ABC294640 resulted in a significant decline in the viable cell population paralleled by a marked increase in early apoptotic cells, especially after a 48-h treatment, in comparison with a single-agent PLX4032 treatment (Figure 5a, Supplementary Figure S4).

We next evaluated the effect of the combination treatment on the migratory capacity of resistant RKOr cells by wound healing assay (Figure 5b). In this assay, the wound width was measured at 0, 6, 12, 24 and 48 h, and the relative wound area was calculated as the ratio of the residual wound area at a given time point and the original wound area at 0 h. Single-agent treatments with either PLX4032 or ABC294640 induced a profound increase in the relative wound area after 24 h in comparison with untreated cells (Figure 5b, Supplementary Figure S5) indicating their inhibitory effect on the migration of resistant cells. However, a more potent anti-migratory effect was achieved with combination treatments of PLX4032 and ABC294640 which evoked a significant increase in the relative wound area as early as 24 h after the treatment when compared to either drug alone, pointing to a strong inhibition of resistant cell migration.

To further investigate whether combined treatments could effectively supress long-term survival of resistant cells, we carried out a colony formation assay. After one week of growth in drug-free media, relative colony formation was assessed as the ratio of the average number of colonies in treated versus untreated cells. Treatment with single-agents PLX4032 and ABC294640 reduced clonogenic growth of resistant cells relative to untreated control at both time points (Figure 5c, Supplementary Figure S6). Importantly, combination treatment with 1.5 µM PLX4032 and 25 µM ABC294640 markedly reduced the colony forming ability of resistant cells after 24 h in comparison with either drug alone. Modest inhibition of colony formation in resistant cells was also observed after concomitant exposure to PLX4032 and ABC294640 at their higher and lower concentrations, respectively, after 12 h when compared to individual drugs (Figure 5c, Supplementary Figure S6).

In short, the obtained results suggest that the combination treatment with SphK2 inhibitor ABC294640 and vemurafenib is more efficient at inducing apoptosis and supressing migratory and clonogenic ability of vemurafenib-resistant cells than a single-agent vemurafenib treatment.

### 2.6. Synergistic Cytostatic Effect of PLX4032 and ABC294640 in Vemurafenib-Resistant Colon Cancer Cells Is Associated with Inhibition of the Regulators of Centrosomal Activity and Mitotic Progression

**Figure 6:**
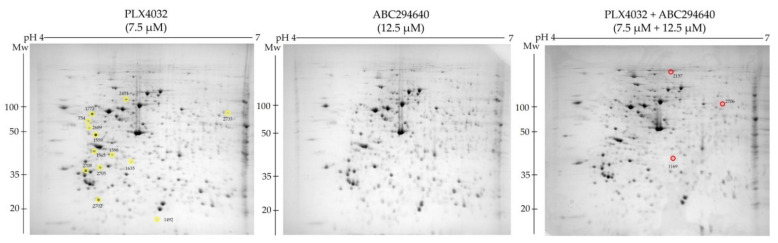
Representative 2-DE gel images of total cell lysates obtained in the pH range 4–7 (7cm IPG strips) from RKOr cells treated with either individual drugs or their combination. Experiments were performed in four biological replicates for each treatment condition. Yellow circles denote protein spots that were significantly decreased in combined treatment relative to each single-drug treatment, while red circles indicate significantly upregulated protein spots.

To explore the molecular mechanisms underlying the increased sensitivity of resistant cell line RKOr to co-treatment of PLX4032 and ABC294640 at the cellular proteome level, we performed comparative proteomic analysis of the cells treated with either single agents ABC294640 (12.5 µM) and PLX4032 (7.5 µM) or their combination for 72 h. Total cell lysates were resolved by two-dimensional polyacrylamide gel electrophoresis (2-DE) on 7 cm IPG strips (pH range 4–7) followed by gel image analysis (Figure 6). We detected 13 and 3 down- and up-regulated protein spots, respectively, with statistical significance (*p* < 0.05) in combination treatment when compared to individual drugs. The identity of the selected protein spots was revealed by MALDI-TOF/TOF mass spectrometric analysis (Table 3).

**Figure 7:**
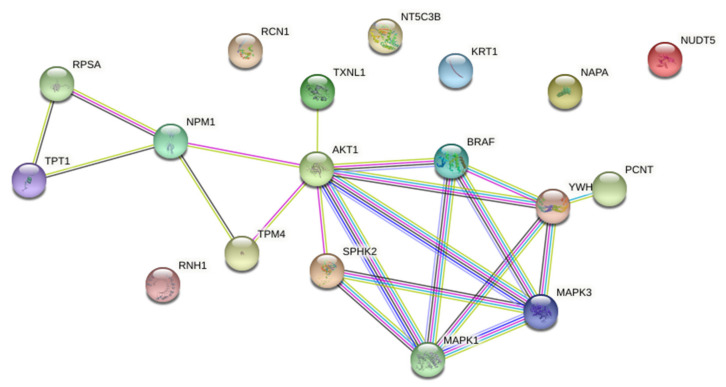
Protein-protein interaction (PPI) network of down-regulated proteins in resistant RKOr cells after combined treatment with ABC294640 and PLX4032 enriched with BRAF, SPHK2, ERK1/2 and AKT1. The PPI network was constructed using the STRING database of known and predicted protein-protein interactions with highest confidence of 0.400.

Functional associations between down-regulated proteins were further analysed by STRING on-line platform (Version: 11.0). Protein-protein interaction (PPI) network was constructed from down-regulated proteins and enriched with AKT and ERK1/2 as known biomarkers of acquired resistance to PLX4032 in BRAFV600E mutant CRC (Supplementary Figure S2), and BRAF and SPHK2 as specific pharmacological targets of PLX4032 and ABC294640, respectively. The PPI network consisted of 18 nodes and 21 edges with the average local clustering coefficient of 0.547 and PPI enrichment *p*-value 0.00649, which suggested that these proteins were at least partially biologically connected as a group (Figure 7).

STRING analysis indicated that AKT was centrally positioned in the network and seemed important in connecting two distinct protein clusters. The first one contained SPHK2, PCNT (pericentrin) and the proteins belonging to the BRAF–MEK–ERK signalling. The second cluster contained four proteins, namely TPM4 (tropomyosin alpha-4 chain), NPM1 (nucleophosmin), RPSA (40S ribosomal protein SA) and TPT1 (translationally-controlled tumour protein; shortly TCTP). Importantly, these two clusters appeared to have a common biological function related to centrosome biology and cell division, in particular centrosome assembly (PCNT), regulation of centrosome duplication (NPM1) and mitotic progression (TCTP).

**Figure 8:**
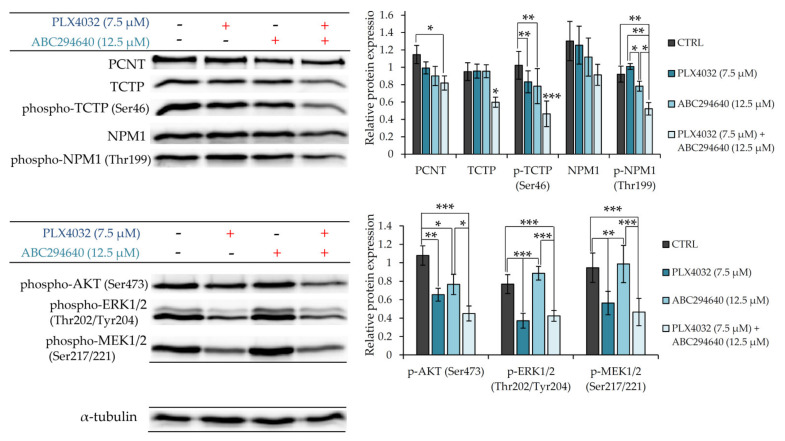
Western blot validation of major proteomic findings indicating the involvement of proteins regulating centrosome biology and cell division in chemosensitizing effect of combination treatment with ABC294640 and PLX4032. Resistant RKOr cells were treated with either PLX4032 (7.5 µM), ABC294640 (12.5 µM) or their combination for 72 h. Relative protein expression was measured using Quantity One software for densitometry analysis of western blot bands. Data were obtained from two independent biological experiments performed in technical duplicates. α-tubulin was used as loading control. Statistical significance is denoted with an asterisk (* *p* < 0.05, ** *p* < 0.01, *** *p* < 0.001). PCNT (Pericentrin), TCTP1 (Translationally-Controlled Tumour Protein), NPM1 (nucleophosmin).

To further verify the involvement of PCNT, NPM1 and TCTP in the mechanisms underlying chemosensitizing effect of combination treatment with ABC294640 and PLX4032, we measured their expression levels by western blot in resistant RKOr cells treated with individual drugs or their combination for 72 h (Figure 8). Combination treatment with PLX4032 and ABC294640 reduced the levels of PCNT in comparison with single-agent treatments. Additionally, a significant decline in the expression levels of TCTP and phospho-TCTP (Ser46) was observed after the co-exposure to ABC294640 and PLX4032, while treatment with either drug alone exhibited only marginal effects on the abundance and activity of TCTP. Finally, co-treatment with ABC294640 and PLX4032 curtailed the level of NPM1 and dramatically diminished the expression of phospho-NPM1 (Thr199) when compared to treatments with either drug alone (Figure 8). An important role of AKT in the mechanisms governing the chemosensitizing effect of combination treatment was additionally corroborated by showing significantly reduced expression levels of its active form phospho-AKT (Ser473) in combined treatment when compared to individual drugs, whereas such an effect was not observed with ERK1/2 and MEK1/2 (Figure 8).

Taken together, these findings implicate that the chemosensitizing effect of combination treatment with ABC294640 and PLX4032 in resistant cells could be, at least partially, ascribed to down-regulation of AKT-mediated pro-survival signalling and suppression of the regulators of centrosomal activity and mitotic progression.

## 3. Discussion

The present study seeks to address the role of bioactive sphingolipid species and the enzymes regulating their metabolism and signalling in the development of resistance to BRAF inhibition by vemurafenib in BRAFV600E mutant colon cancer cells. It has previously been shown that BRAF mutated RKO colon cancer cells have remarkably higher activities and protein expression levels of SphK1 and SphK2 in comparison to several other colon cancer cell lines harbouring wild-type BRAF, and that they exhibit the least sensitivity to oxaliplatin [4]. As expected, the treatment of RKO cells with a dual SphK1/SphK2 inhibitor significantly increased cytotoxic effects of oxaliplatin, which suggests that SphK1 and SphK2 regulate chemosensitivity of BRAF mutant colon cancer cells. Similarly, we found increased baseline levels of activated forms of sphingosine kinases 1 and 2 in BRAF mutated colon cancer cells resistant to vemurafenib in comparison with parental cells. Moreover, vemurafenib exposure drastically up-regulated expression levels of active forms of both sphingosine kinases in resistant cells relative to their sensitive counterparts, which indicates that vemurafenib may potentially contribute to a metabolic shift towards the production of S1P in resistant cells. In line with this, mass spectrometry-based sphingolipidomics analyses revealed markedly increased levels of S1P and its precursor sphingosine in resistant cells under both basal conditions and upon treatment with vemurafenib.

Besides serving as a precursor for S1P production, sphingosine can be recycled in the sphingolipid salvage pathway to generate ceramides. Our results revealed an upsurge in the levels of long-chain ceramide 16:0 and very long-chain ceramides 22:0/24:0 produced by CerS6 and CerS2, respectively, in resistant vs. sensitive cells under basal conditions and after the vemurafenib challenge. Furthermore, the involvement of the ceramide salvage pathway in the mechanisms underlying vemurafenib resistance in BRAF mutant colon cancer cells was also detected by western blot analyses that showed an increased expression of ceramide-producing enzymes CerS2 and CerS6 as well as an up-regulation of ceramide-degrading enzyme ASAH1 in resistant cells under basal conditions and particularly after the exposure to vemurafenib. However, the non-responsiveness of colon cancer cells to vemurafenib also seems to include the mechanisms that regulate de novo ceramide synthesis pathway, as indicated by a marked rise in the baseline levels of sphinganine in resistant cells. This pathway can be metabolically triggered by metabolic loading with serine, whose increased biosynthesis pathway at the enzyme level has previously been shown to be a distinctive metabolic feature of BRAFV600E mutant colon cancer cells associated with the development of resistance to vemurafenib [15,16]. Our data suggest that the acquired resistance to vemurafenib could be, at least partially, associated with the induction of distinct pathways of ceramide formation whose differential activation is possible due to spatial separation of the enzymes regulating ceramide generation [17]. Notably, the long-chain ceramide 16:0 and very long-chain ceramides 22:0/24:0 could play cytoprotective roles by conferring a growth advantage to resistant cells, which is in good agreement with previous studies showing tumour growth-promoting abilities of C16- and C24-ceramides in different cancer types including colon cancer [18,19,20]. Accordingly, C16:0 and C22:0/24:0 ceramides should be further studied as potential resistance biomarkers, and their potential benefits in monitoring therapeutic efficacy of vemurafenib in BRAF mutant colon cancer should be further explored.

Since the obtained data clearly pointed to increased regulation of S1P production and a metabolic shift favouring the production of specific long-chain and very long-chain ceramide species as molecular features of acquired resistance to vemurafenib, we hypothesized that either inhibiting sphingosine kinases 1 and 2 or retuning the ceramide balance by exogenous short-chain C6 ceramide could restore vemurafenib sensitivity in resistant BRAF mutant colon cancer cells. Indeed, the pharmacological inhibition of SphK1 and SphK2 activities by selective inhibitors PF-543 and ABC294640 (Opaganib), respectively, was achieved, as well as the incubation with C6-ceramide augmented cytostatic effect of vemurafenib in resistant cells in a synergistic manner, even when low sub-toxic concentrations were applied. Importantly, the combination of ABC294640 and vemurafenib seems the most promising for further research due to their lowest cytotoxicity, the largest number of synergistic drug combinations in low sub-IC_50_ concentration range and available safety and efficacy of the data from completed [21] and on-going clinical studies in hematological malignancies (NCT02229981, NCT02757326) and hepatocellular carcinoma (NCT02939807). Moreover, the potent synergistic anti-proliferative effect of the combination treatment with ABC294640 and vemurafenib was confirmed in two different BRAF mutant colon cancer cells lines RKO and HT-29 with acquired resistance to vemurafenib, which supports the potential therapeutic value of this drug combination in combating drug resistance in BRAF mutant colon cancer.

ABC294640 was previously shown to exert a strong anti-proliferative activity in BRAFV600E mutant HT-29 colon cancer cell lines in a dose- and time-dependent manner and to decrease the colony forming ability and induce apoptosis in HT-29 cells [6]. Similarly, we have shown that the synergistic anti-cancer effect of the combination treatment with ABC294640 and vemurafenib in resistant RKO cells resulted in markedly increased apoptosis and reduced migration and clonogenic capacity in comparison with single-agent vemurafenib. This could be, at least partially, attributed to ABC294640-induced inhibition of sphingosine kinase activity and a consequent reduction in S1P concentration, as previously described in HT-29 cells cultured in the presence of cytotoxic concentrations of ABC294640 [6]. Our data revealed that a co-treatment with vemurafenib and ABC294640 significantly reduced cellular levels of S1P in comparison with either drug alone, which again confirms that the aberrant regulation of S1P metabolism might be a hallmark of drug resistance in BRAF mutant colon cancer cells. A decline in the S1P content in resistant cells induced by combined treatment concurred with a significant reduction in sphinganine levels is suggestive of a suppression of de novo sphingolipid biosynthesis pathways.

Additional proteomic study supported by western blot validation provided some novel insights into the molecular and cellular events concurring with the chemosensitizing effect of ABC294640 in combination with PLX4032 in vemurafenib-resistant RKO colon cancer cells. The obtained results showed that a combination treatment significantly reduced expression levels of several proteins involved in centrosome-associated functions related to cell cycle regulation and microtubule organization, mitosis and proliferation including pericentrin (PCNT), nucleophosmin (NPM1) and translationally-controlled tumour protein (TCTP). Pericentrin is an integral component of the centrosome that regulates centrosome organization and spindle assembly, and has therefore been considered a reliable marker for centrosomes and an acentriolar microtubule organizing centre [22]. Similarly, nucleophosmin has been identified as a constituent of the centrosome that regulates the initiation of centrosome duplication. Specifically, nucleophosmin associates with unduplicated centrosomes; however, CDK2/cyclin E-mediated phosphorylation of nucleophosmin on threonine 199 promotes its dissociation from the centrosomes and enables initiation of centrosome duplication [23]. Previously, we found significantly increased abundance of cytoplasmic *p*-NPM1 (Thr199) in tumour tissue from BRAF-mutated in comparison with wild-type BRAF colon adenocarcinoma patients and demonstrated the role of p-NPM1 (Thr199) in mediating the resistance to vemurafenib in BRAF mutant colon cancer cells [16]. In the present study, we showed a significant decline in the expression levels of phospho-NPM1 (Thr199) in resistant RKO cells grown in the presence of ABC294640 and vemurafenib in comparison to either drug alone. The study in mouse embryonic stem cells has shown that TCTP forms a complex with NPM1 which peaks sharply during mitosis and promotes cell proliferation [24]. TCTP facilitates mitotic cell division by stabilising the mitotic spindle. TCTP binds to the mitotic spindle but is detached from the spindle during metaphase-anaphase transition [25]. Phosphorylation of TCTP at serine 46 by Plk-1 facilitates the detachment of TCTP from the spindle, which enables progression through mitosis. Our results revealed that the expression level of phospho-TCTP (Ser46) was significantly reduced when resistant RKO cells were cultured in the presence of a combination treatment as compared to the use of individual drugs. In the light of the obtained proteomic and western blot data, it is likely that the potent growth-inhibitory effect of concomitant exposure to ABC294640 and vemurafenib in resistant cells is linked with a reduced proliferation capacity due to the suppression of molecular events that regulate centrosome function and assembly as well as mitotic progression.

Several lines of evidence have demonstrated that low cytotoxic concentrations of ABC294640 markedly supress AKT phosphorylation at both Ser473 and Thr308 in HT-29 cells [6]. Since S1P specifically induces AKT phosphorylation via the extracellular S1P pathway to promote cell proliferation and enable cell survival [26], anti-cancerous effects of ABC294640 could be ascribed to attenuation of S1P-mediated AKT activation resulting in cell growth suppression. The involvement of increased regulation of AKT activity in drug resistance in BRAF mutant colon cancer was previously confirmed by higher basal levels of phospho-AKT in RKO cells intrinsically resistant to oxaliplatin in comparison with the responsive HCT116 colon cancer cells [4]. While sensitive HCT116 cells reduced their levels of p-AKT upon the exposure to oxaliplatin, RKO cells cultured in the presence of oxaliplatin exerted sustained AKT phosphorylation. Both the treatment with oxaliplatin and pharmacological inhibitor of SphK activity, and SphK1 or SphK2 knockdown in RKO cells treated with oxaliplatin, resulted in a dramatic decline in AKT phosphorylation levels accompanied by the induction of apoptosis [4]. Similarly, our data demonstrated that the co-treatment with ABC294640 and vemurafenib gave rise to a significant decline in the expression level of phospho-AKT (Ser473) in comparison with single-agent treatments, which indicates that the synergistic cytostatic effect of a combination treatment in vemurafenib-resistant cells could be, at least partially, attributed to the abrogation of S1P-induced activation of pro-survival AKT signalling pathway.

An important role of AKT in mediating chemosensitivity to combined treatment with ABC294640 and vemurafenib was also supported by bioinformatics analysis, which revealed direct and indirect functional associations between AKT and the proteins regulating centrosome biology and mitotic progression, namely NPM1 and TPT1 (TCTP). The clue that NPM1 and AKT work together to promote proliferation and survival of BRAFV600E mutant colon cancer cells came from a previous study showing that either pharmacological inhibition of NPM1 function by NSC348884 at a sub-toxic concentration or NPM1 knockdown strongly potentiated anti-proliferative effects of standard chemotherapeutic agents and augmented apoptosis induction in RKO cells, which was accompanied by diminished expression levels of phospho-AKT (Ser473) [27]. Importantly, the treatment of RKO cells expressing high endogenous levels of NPM1 with the inhibitor of the PI3K/AKT pathway significantly enhanced cell sensitivity to 5-fluorouracil, which suggests that AKT signalling participates in regulating the oncogenic and anti-apoptotic effects of NPM1 expression that reduce the efficacy of chemotherapy in BRAF mutant colon cancer.

The anti-apoptotic protein TCTP has been previously reported to contribute to non-responsiveness of colon cancer cells to chemotherapy agents [28]. Knockdown of TCTP suppresses proliferation, migration and invasion capacity of colon cancer cells in vitro and in vivo [29]. In addition, extracellular TCTP was shown to induce cell migration and invasion of colon cancer cells in vitro supporting its metastasis-promoting role in colon cancer [30]. Importantly, the growth factor-dependent induction of TCTP protein expression levels in HT-29 cells could be dramatically reduced by pharmacological inhibition of AKT, which indicates that TCTP synthesis is regulated by the AKT pathway in BRAF mutated HT-29 colon cancer cells [31].

**Figure 9:**
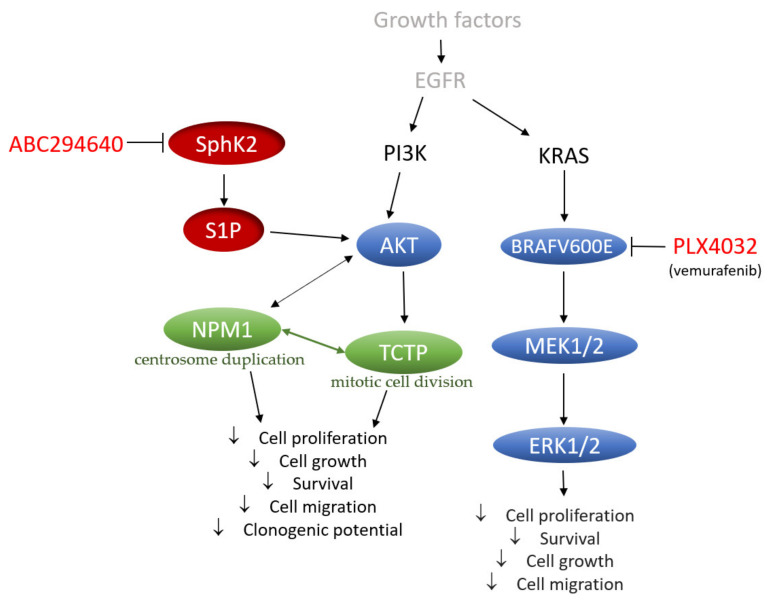
The proposed mechanisms underlying acquired resistance to vemurafenib in BRAFV600E mutant colon cancer cells including known (blue) and newly identified (red) features. Potential novel targets of combination treatment with ABC294640 and vemurafenib whose down-regulation concurs with chemosensibilization effect in vemurafenib-resistant RKO colon cancer cells are also shown (green). EGFR (epidermal growth factor receptor); SphK2 (sphingosine kinase 2); S1P (sphingosine-1-phosphate); NPM1 (nucleophosmin); TCTP (translationally-controlled tumour protein).

In conclusion, our data provide a novel perspective on the mechanisms underlying the acquired resistance to vemurafenib in BRAFV600E mutant colon cancer cells that include increased regulation of SphK-catalysed S1P production and altered ceramide metabolism, in particular up-regulation of the ceramide salvage and de novo sphingolipid synthesis pathways (Figure 9). The pharmacological inhibition of SphK2 by ABC294640 (Opaganib) in the studied RKO and HT-29 vemurafenib-resistant cell models has proved effective at restoring the sensitivity of resistant cells to vemurafenib across multiple combinations of sub-toxic drug concentrations, while exerting low cytotoxicity. The reduced proliferation, migratory and clonogenic ability and increased apoptosis in resistant RKO cells induced by the combination treatment as compared to single-agent vemurafenib were accompanied by the attenuation of S1P-regulated AKT activity, which may contribute to abrogation of AKT-mediated cellular effects of nucleophosmin and translationally-controlled tumour protein (Figure 9). Thus, multi-drug nature of the proposed novel therapeutic strategy could contribute to increased treatment response in BRAF mutated colon cancer cells. The available safety and toxicity data on ABC294640 from on-going clinical trials additionally justify further preclinical studies to explore potential clinical benefits of co-administering ABC294640 and vemurafenib in treating colon cancer patients harbouring the BRAFV600E mutation.

## 4. Materials and Methods

### 4.1. Cell Culturing and Development of Vemurafenib-Resistant Colon Cancer Cell Lines

Human colon carcinoma cell lines RKO and HT-29 (BRAFV600E/KRAS*wt*) were purchased from the ATCC and maintained in Dulbecco′s Modified Eagle′s medium (DMEM) or Minimum Essential Medium (MEM) supplemented with 10% foetal bovine serum (FBS), 2 mM L-glutamine, penicillin (100 U/mL) and streptomycin (100 µg/mL) (Capricorn Scientific, Ebsdorfergrund, Germany) in humified atmosphere with 5% CO_2_ at 37 °C.

In order to eliminate molecular features of resistance that might be cell-line specific, we developed two vemurafenib (PLX4032)-resistant colon cancer cell lines derived from HT-29 and RKO cell lines by exposing the cells to successively increasing concentrations of PLX4032 (MedChemExpress, Monmouth Junction, NJ, USA) in the period of about 6 months until clinically relevant dose (11.52 µM) [7] was reached. Established resistance phenotypes were confirmed by the MTT assay showing an increase in the IC_50_ values by 10- and 6.7-fold in the resistant RKO and HT-29 cells, respectively, in comparison with their sensitive counterparts (Supplementary Table S1).

### 4.2. Western Blot Analysis

Cells were seeded in 6-well plates at a density of 1.5 × 10^5^ cells per well and cultured for indicated time period in the presence or absence of test agent. Cells were then lysed using RIPA buffer (25 mM Tris-HCl (pH 7.4), 1% NP-40, 0.5% Sodium Deoxycholate, 0.1% SDS, 150 mM NaCl (Sigma-Aldrich, St. Louis, MO, USA)) supplemented with protease and phosphatase inhibitor cocktails (Roche, Basel, Switzerland). A total of 50 µg proteins were resolved on 7% or 12% SDS polyacrylamide gels and transferred onto PVDF membrane (Bio-Rad, Hercules, CA, USA). Membranes were blocked in either 5% bovine serum albumin (BSA) or non-fat milk prepared in TBST and probed with primary antibodies against phospho-SphK1 (Ser225), phospho-S1P1 (Thr236), EDG3, Lass6, Lass2, ASM and NSmase1 and neutral ceramidase (ASAH2) from Thermo Fisher Scientific (Waltham, MA, USA), acid ceramidase (ASAH1) from Santa Cruz Biotechnology (Dallas, TX, USA) and phospho-SphK2 (Thr578) (Abcam, Cambridge, UK) all in concentration 1:1000. Furthermore, NPM1 (1:4000) and SGPL1 (1:1000) from Sigma-Aldrich (St. Louis, MO, USA) and phospho-NPM1 (Thr199), PCNT, TCTP, phospho-TCTP (Ser46) phospho-ERK1/2 (Thr202/Tyr204), phospho-MEK1/2 (Ser217/221), phospho-AKT (Ser473) and α-tubulin (1:1000) from Cell Signalling Technologies (Danvers, MA, USA), overnight at 4 °C. The next day, membranes were washed with TBST and probed with secondary antibody goat, anti-mouse, or goat anti-rabbit (Cell Signalling Technologies, 1:2000). Protein bands were visualized using Amersham™ ECL™ Prime Western Blotting Detection Reagent and Imagequant LASS 500 (GE Healthcare, Chicago, IL, USA). Relative protein expression was analysed by Quantity One 1-D Analysis Software (Bio-Rad, Hercules, CA, USA). Statistical analysis was performed by the two-tailed t-test, where *p*-value < 0.05 was considered statistically significant.

### 4.3. Cell Viability Assay

Cell viability was assessed using the MTT assay. Briefly, cells were seeded onto 96-well microtiter plates at a seeding density of 3000 cells/well. Following day, cells were treated with test agents in five 10-fold serial dilutions (10^−4^–10^−8^ µM) and further incubated for 72 h. MTT assay was performed according to the manufacturer’s instructions (Sigma-Aldrich, St. Louis, MO, USA). In brief, after the completion of treatment period, cells were incubated with MTT reagent for 3 h in the dark followed by the addition of dimethyl sulfoxide (DMSO, Sigma-Aldrich, St. Louis, MO, USA). Absorbance was measured at 570 nm using Sunrise Absorbance microplate reader (Tecan Life Sciences, Männedorf, Switzerland). Inhibitory and lethal concentrations (IC_50_ and LC_50_, respectively) were calculated using linear regression analysis.

Combined treatments were performed using five 2-fold serial dilutions of PLX4032 IC_50_ concentrations (30, 15, 7.5, 3, 1.5 µM) in combination with three 2-fold dilutions of IC_50_ concentrations of ceramide C6 (5, 2.5, 1.25 µM), or PF-543 (30, 15, 7.5 µM) or ABC294640 (50, 25, 12.5 µM). All inhibitors are commercially available and were purchased from MedChemExpress (MedChemExpress, Monmouth Junction, NJ, USA). After treatment for 72 h cell viability was assessed using the MTT assay as described above. Combination index (CI) was calculated using CompuSyn software [32]. Combination index (CI) values that correspond to <1, =1 and >1 indicate on synergy, additivity, and antagonism, respectively. Each experiment was performed as tetraplicate in three independent biological experiments.

### 4.4. Detection of Apoptosis Using Annexin V Assay

Detection of apoptosis was carried out by using Annexin-V FLUOS Staining kit (Roche, Basel, Switzerland). Briefly, 2 × 10^4^ cells were seeded in 8 well chambers (Nunc^®^ Lab-Tek^®^ Chamber Slide™ system, Sigma-Aldrich, St. Louis, MO, USA). The other day, cells were treated with compounds in selected concentrations and incubated for further 24 and 48 h. Media was removed and cells were washed with PBS. Further steps were performed according to the manufacturer’s instructions. Slides were analysed under fluorescent microscope (Zeiss, Oberkochen, Germany) using 10x magnification.

### 4.5. Wound Healing Assay

In brief, cells were seeded into 6-well plates at density of 1.5 × 10^5^ cells per well. Second day, a scratch was made across the well using a yellow pipette tip (2–200 µL) and cells were treated as indicated. Immediately after treatment, wound area was imaged using a phase contrast microscope (Zeiss, Oberkochen, Germany) for 0-h treatment point. The wound was monitored further for 6, 12, 24 and 48 h. Wound width was measured using Image J software (National Institutes of Health, MD, USA) in three different spots for each time point in two individual biological experiments in technical replicate.

### 4.6. Clonogenic Assay

Cells were seeded into 6-well plates and treated for 12 and 24 h with determined concentrations of inhibitors. After treatment, cells were passaged and seeded into new 6 well plates in concentration of 500 cells per well and cultured for 7 days under agent-free conditions. Formed cell colonies were fixed using acetic acid and methanol (1:7) and stained with 0.5% crystal violet (Sigma-Aldrich, St. Louis, MO, USA). Only colonies that contained more than 50 cells were counted.

### 4.7. Sphingolipid Analysis by Mass Spectrometry

Cell pellets (~2 × 10^6^ cells) were transferred to 2 mL Safe-Lock PP-tubes and lipids were extracted according to Matyash et al. [33]. In brief, samples were homogenized using two 6 mm steal beads on a Mixer Mill (Retsch, Haan, GER; 2 × 10 sec, frequency 30/s) in 700 µL MTBE/MeOH (3/1, *v*/*v*) containing 500 pmol butylated hydroxytoluene and 1% acetic acid as additives as well as ceramide d18:1/17:0 (1.95 pmol), sphingomyelin 18:1/17:0 (7.5 pmol), sphingosine-1-phosphate-d7 18:1 (6 pmol), and sphingosine-d7 (1.2 pmol) as internal standards (IS, Avanti Polar Lipids, Alabaster, AL, USA). Total lipid extraction was performed under constant shaking for 30 min at RT. After addition of 140 µL dH2O samples were vigorously vortexed (3 × 10 sec) and centrifuged at 1000× *g* for 15 min. 500 µL of the upper, organic phase were collected and dried under a stream of nitrogen. Lipids were resolved in 200 µL 2-propanol/methanol/dH2O (7/2.5/1, *v*/*v*/*v*) and 6 µL were injected for UHPLC-QqQ analysis. Residual protein slurry was dried and used for BCA protein determination after lysis in 200 µL NaOH (0.3N) at 60 °C for 4 h. Chromatographic separation was performed on an 1290 Infinity II LC system (Agilent, Santa Clara, CA, USA) equipped with Zorbax RRHD Eclipse Plus C18 column (2.1 × 50 mm, 1.8 µm; Agilent, Santa Clara, CA, USA) running a 10 min gradient from 60% solvent A (H2O; 10 mM ammonium acetate, 0.1% formic acid, 8 µM phosphoric acid) to 100% solvent B (2-propanol; 10 mM ammonium acetate, 0.1% formic acid, 8 µM phosphoric acid) at 500 µL/min flow rate (Table 4). The column compartment was kept on 50 °C. A 6470 triple quadrupole mass spectrometer (Agilent, Santa Clara, CA, USA) equipped with an ESI source was used for detection of lipids in positive mode (Table 5). Lipid species were analyzed by dynamic multiple reaction monitoring (ceramides [M + H] + [–H_2_O] to *m*/*z* 264.2, CE 24, Fragmentor 199, CAV 5; sphingomyelin [M + H]+ to *m*/*z* 184.1, CE 24, Fragmentor 184, CAV 5; sphingosine-1-phosphate *m*/*z* 380.3 to *m*/*z* 264.2, CE 16, Fragmentor 75, CAV 5; sphingosine *m*/*z* 300.3 to *m*/*z* 282.2, CE 8, Fragmentor 78, CAV 5). Data acquisition and data processing was done by MassHunter Data Acquisition software (Version 10.0 SR1, Agilent, Santa Clara, CA, USA) and MassHunter Workstation Quantitative Analysis for QQQ (Version 10.0, Agilent, Santa Clara, CA, USA) respectively. Data were normalized for recovery, extraction-, and ionization efficacy by calculating analyte/IS ratios and expressed as fmol/µg protein.

### 4.8. Two-Dimensional Gel Electrophoresis and Image Analysis

Cells were lysed in 2-DE lysis buffer containing 7M urea, 2M thiourea, 4% CHAPS and 1% DTT (Sigma-Aldrich, St. Louis, MO, USA) supplemented with protease inhibitor cocktail (Roche, Basel, Switzerland). A total of 150 µg of proteins was solubilized in 2-DE rehydration buffer (7M urea, 2M thiourea, 4% CHAPS, 1% DTT and 0.2% Bio-Lyte ampholyte (Bio-Rad, Hercules, CA, USA), loaded onto 7 cm pH 4–7 IPG strips and subjected to isoelectric focusing on PROTEAN IEF cell (Bio-Rad, Hercules, CA, USA). The IEF conditions were as follows: 50 V for 12 h, 250 V for 15 min, 250–4000 V for 1 h and 4000 V for 4 h. In the second dimension, proteins were resolved by 12% SDS-polyacrylamide gels by Mini-PROTEAN Tetra Cell (Bio-Rad, Hercules, CA, USA). Gels were stained in Coomassie Blue G-250 (Sigma-Aldrich, St. Louis, MO, USA) overnight, and after washing in miliQ water, gel images were taken by ChemiDoc XRS+ Imager (Bio-Rad, Hercules, CA, USA). 2-DE gel image analysis was carried out using Progenesis SameSpots 4.0 software (TotalLab, Newcastle upon Tyne, UK). The experiment was performed in four individual biological replicates for each condition. ANOVA analyses followed by post hoc Tukey’s test were carried out to identify statistically significant differences in protein abundance between the datasets obtained for the three different treatment regimens.

### 4.9. MALDI-TOF/TOF Mass Spectrometry Analysis

Each sample was mixed with matrix solution containing α-cyano-4-hydroxycinnamic acid (0.3 g/L CHCA in a solution containing 2:1 ethanol:acetone, *v*/*v*) at the ratio of 1:10. A total amount of 1 µL of the mixture containing sample/matrix solution was spotted onto the MALDI plate (AnchorChip 800 μm, Bruker Daltonics, Bremen, Germany) and kept at room temperature to allow crystallization to occur. UltrafleXtreme MALDI-TOF/TOF mass spectrometer (Bruker Daltonics, Billerica, MA, USA) was used to perform MS analyses in the reflector mode in the m/z range of 700–3500 Da. The MS spectra were externally calibrated with the mixture of Peptide Calibration Standard and Protein Calibration Standard I (Bruker Daltonics, Billerica, MA, USA) at the ratio of 1:5. FlexControl 3.4 software (Bruker Daltonics, Billerica, MA, USA) was applied to acquire and process spectra. FlexAnalysis 3.4 (Bruker Daltonics, Billerica, MA, USA) was applied to perform protein database searches. Proteins were identified using the Mascot 2.4.1 search engine (Matrix Science, London, UK). The following search parameters were applied: Enzyme: trypsin; Fixed modifications: Carbamidomethylation on cysteine; Variable modifications: Oxidation on methionine; Protein mass: Unrestricted; Peptide mass tolerance: ±50 ppm; Maximum missed cleavage: 2.

### 4.10. Bioinformatic Analyses

Search Tool for retrieval of Interacting Genes (STRING) (http://string-db.org/, accessed 1 June 2021) online tool was applied to construct the PPI network, where the confidence score was set to 0.400. 

## Figures and Tables

**Table 1:** IC_50_ and LC_50_ values of tested agents obtained by the MTT assay in resistant RKOr cell line.

		C6-Ceramide		
		5 µM	2.5 µM	1.25 µM
**PLX4032**	IC_50_ (µM)	<5	<2.5	8.64 ± 1.01
	LC_50_ (µM)	>5	>2.5	>1.25
		**PF-543**		
		**30** µM	**15** µM	**7.5** µM
**PLX4032**	IC_50_ (µM)	2.56 ± 0.39	11.27 ± 1.25	17.77 ± 2.05
	LC_50_ (µM)	>30	>15	>7.5
		**ABC294640**		
		**50** µM	**25** µM	**12.5** µM
**PLX4032**	IC_50_ (µM)	<50	4.99 ± 0.88	13.28 ± 1.53
	LC_50_ (µM)	24.04 ± 2.03	>25	>12.5

**Table 2:** Combined therapeutic effects of either C6 ceramide, sphingosine kinase 1 inhibitor PF-543 or sphingosine kinase 2 inhibitor ABC294640 and vemurafenib in vemurafenib-resistant RKO colon cancer cell line. The combination index (CI) was calculated for each drug combination, where CI < 1 indicates synergy; CI = 1 indicates additivity; and CI > 1 indicates antagonistic effects. Drug combinations exhibiting synergistic effect were shown in bold.

C6 (µM)	PLX4032 (µM)	CI	PF-543 (µM)	PLX4032 (µM)	CI	ABC294640 (µM)	PLX4032 (µM)	CI
**1.25**	**1.5**	**0.62**	7.5	7.5	1.40	12.5	1.5	1.22
1.25	5	1.41	**7.5**	**15**	**0.88**	12.5	3	1.15
1.25	7.5	1.61	15	1.5	1.78	**12.5**	**7.5**	**0.89**
1.25	15	1.43	15	3	1.68	**12.5**	**15**	**0.69**
**2.5**	**1.5**	**0.64**	15	7.5	1.01	**12.5**	**30**	**0.43**
2.5	5	1.13	**15**	**15**	**0.87**	**25**	**1.5**	**0.88**
			30	1.5	1.34	**25**	**3**	**0.90**
			30	3	1.11	**25**	**7.5**	**0.82**
			**30**	**7.5**	**0.63**	**25**	**15**	**0.74**
						**25**	**30**	**0.43**
						**50**	**1.5**	**0.83**
						**50**	**3**	**0.74**
						**50**	**7.5**	**0.55**

**Table 3:** Down- and up-regulated proteins with statistical significance (*p* ≤ 0.055) in vemurafenib-resistant RKO colon cancer cells cultured in the presence of combined treatment with vemurafenib and ABC294640 in comparison with single-drug treatments with either vemurafenib or ABC294640. Protein identification was carried out by MALDI-TOF/TOF mass spectrometry.

Significantly Down-Regulated in Combined Therapy						
Spot Number	Accession ID	Protein Name *p*-Value	Molecular Weight (kDa)	Peptide Matches	Sequence Coverage (%)	SCORE
**2689**	RCN1_HUMAN	Reticulocalbin-1 (0.0003)	38.87	11	32.00	56
**2702**	TCTP_HUMAN	Translationally-controlled tumor protein (0.004)	19.70	10	43.00	58
**1635**	SNAA_HUMAN	Alpha-soluble NSF attachment protein (0.008)	33.67	22	74.00	137
**2705**	NUDT5_HUMAN	ADP-sugar pyrophosphatase (0.013)	24.60	8	37.00	54
**1559**	RSSA_HUMAN	40S ribosomal protein SA (0.013)	32.95	12	43.00	84
**1565**	NPM_HUMAN	Nucleophosmin (0.013)	32.73	11	34.00	66
**2708**	Mixture: TPM4_HUMAN 1433E_HUMAN	Tropomyosin alpha-4 chain 14-3-3 protein epsilon (0.018)	28.62; 29.33	16; 17	51.00; 64.00	84; 75
**1598**	TXNL1_HUMAN	Thioredoxin-like protein 1 (0.023)	32.63	17	17.00	109
**2733**	K2C1_HUMAN	Keratin, type II cytoskeletal 1 (0.041)	66.17	16	39.00	75
**1492**	5NT3B_HUMAN	7-methylguanosine phosphate-specific 5’-nucleotidase (0.046)	34.65	7	38.00	52
**1773**	KAD1_HUMAN	Adenylate kinase isoenzyme 1(0.073; 0.043)	21.74	8	52.00	60
**2451**	PCNT_HUMAN	Pericentrin (0.053)	380.60	33	14.00	58
**754**	RINI_HUMAN	Ribonucelase inhibitor (0.053)	51.77	14	40.00	69
**Significantly Up-Regulated in Combined Therapy**						
**Spot Number**	**Accession ID**	**Protein Name** ***p*-Value**	**Molecular Weight (kDa)**	**Peptide Matches**	**Sequence Coverage (%)**	**SCORE**
**2706**	SYWC_HUMAN	Tryptophan--tRNA ligase, cytoplasmic (0.009)	53.47	13	31.00	52
**1169**	NAA10_HUMAN	N-alpha-acetyltransferase 10 (0.031)	26.61	12	36.00	57
**2157**	SYAC_HUMAN	Alanine--tRNA ligase, cytoplasmic (0.032)	107.48	27	36.00	92

**Table 4:** LC gradient conditions used in sphingolipid analysis by mass spectrometry.

LC Gradient Timetable				
Time (min)	A (%)	B (%)	Flow (mL/min)	Pressure Limit (bar)
0	60	40	500	850
0.5	60	40	500	850
8.5	0	100	500	850
9.25	0	100	500	850
9.30	60	40	500	850
10	60	40	500	850

**Table 5:** Table showing ESI parameters used in sphingolipid analysis by mass spectrometry.

ESI Parameters	
Gas Temp. (°C)	300
Gas flow (l/min)	5
Nebulizer (psi)	30
Sheath Gas Temp (°C)	400
Sheath Gas Flow (l/min)	12
Capillary (V)	3500
Nozzle (V)	0

## Data Availability

Not applicable.

## Supplementary Materials

The following are available online at https://www.mdpi.com/article/10.3390/ijms221910767/s1.
