# Supplementary material for: The Sphingosine Kinase 2 Inhibitor ABC294640 Restores the Sensitivity of BRAFV600E Mutant Colon Cancer Cells to Vemurafenib by Reducing AKT-Mediated Expression of Nucleophosmin and Translationally-Controlled Tumour Protein
Source: Int J Mol Sci. 2021 Oct 5;22(19):10767. doi: 10.3390/ijms221910767 (PMC8509245; doi:10.3390/ijms221910767)
Supplement: Supplementary file 1 [file ijms-22-10767-s001.zip › ijms-1396190-supplementary.pdf]

Supplementary Material

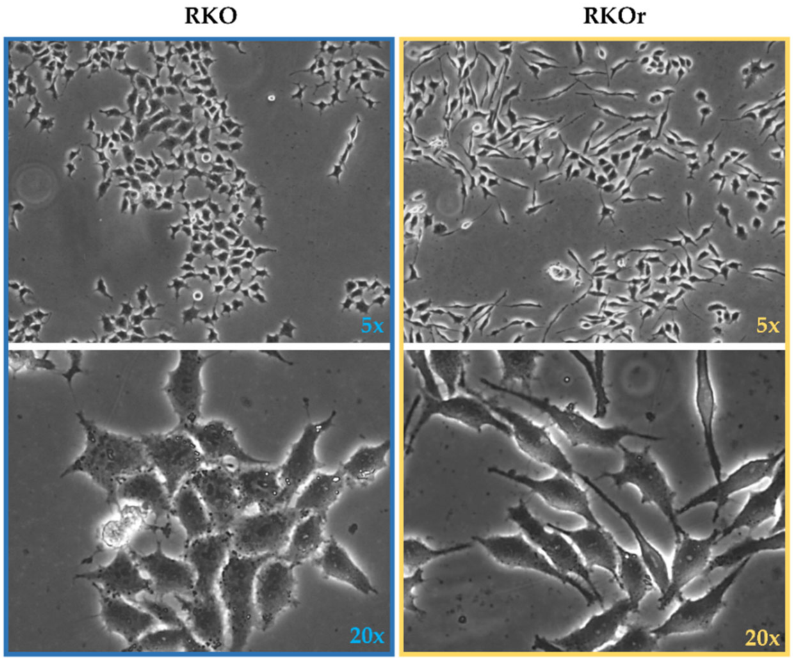

Supplementary Figure S1. Microscopic images depicting distinct morphological features of vemurafenib-resistant RKO colon cancer cells harbouring BRAFV600E mutation in comparison with their sensitive counterpart. Images were taken at 5x and 20x magnification.

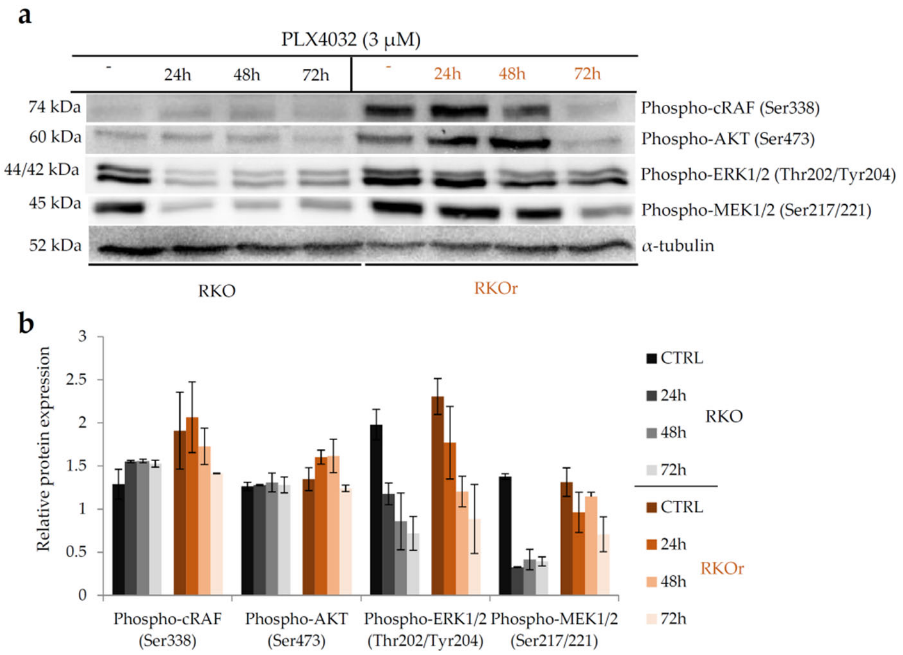

Supplementary Figure S2. Time-dependent changes in relative expression of resistance biomarkers after treatment of RKO and RKOr with PLX4032 (3 μM) for 24, 48 and 72 hours. Figure represents expression patterns of p-cRAF (Ser338), p-AKT (Ser473), p-ERK1/2 (Thr202/Tyr204) and p-MEK1/2 (Ser217/221) major hallmarks of CRC resistance

to vemurafenib. Relative protein expression was measured using Quantity One software for densitometry analysis of Western blot bands to calculate relative abundance of selected proteins in parental RKO and resistant RKOr cells (**a**). Data represent mean and standard deviation obtained from two independent biological experiments (**b**).

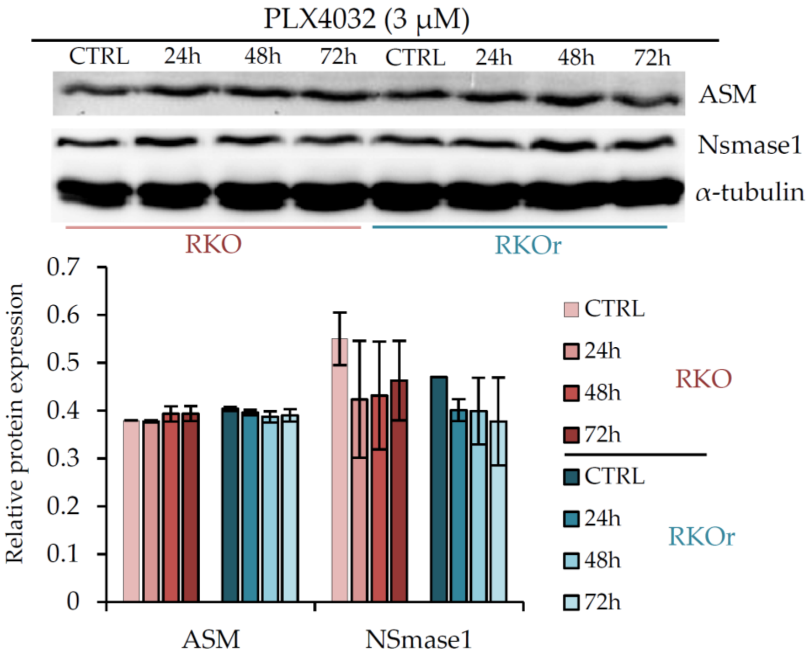

Supplementary Figure S3. Time-dependent changes in relative expression of sphingolipid metabolism mediators acid sphingomyelinase (ASM) and neutral sphingomyelinase 1 (NSmase1) after treatment of RKO and RKOr with PLX4032 (3.0 μM) for 24, 48 and 72 hours. Figure represents expression patterns of enzymes for degradation of sphingomyelin species. Relative protein expression was measured using Quantity One software for densitometry analysis of western blot bands to calculate relative abundance of selected proteins in parental RKO and resistant RKOr cells. α-tubulin was used as a loading control. ASM (acid sphingomyelinase), NSmase1 (neutral sphingomyelinase 1)

Supplementary Table S1. Anti-proliferative effects of PLX4032 in BRAF mutant colon cancer cells and the cells with acquired resistance to PLX4032. Shown here are the results from three independent experiments expressed as mean ± standard deviation.

Inhibitor		RKO (μM)	RKOr (μM)	HT-29 (μM)	HT-29r (μM)
PLX4032	IC_{50}	3.03 ± 1.03	29.76 ± 0.62	5.52 ± 1.47	37.39 ± 1.97
	LC_{50}	49.62 ± 4.71	74.29 ± 2.28	69.98 ± 3.51	84.81 ± 5.24

Supplementary Table S2. Anti-proliferative effects of exogenous ceramide C6, PF-543 and ABC294640 in BRAF mutant colon cancer cells and the cells with acquired resistance to PLX4032. Data are expressed as means ± standard deviation from three independent biological experiments.

Inhibitors		RKO (μM)	RKOr (μM)	HT-29 (μM)	HT-29r (μM)
C6-ceramide	IC_{50}	4.31 ± 1.37	4.78 ± 1.34		
	LC_{50}	>100	>100		
PF-543	IC_{50}	63.37 ± 6.12	31.47 ± 5.75		
	LC_{50}	>100	85.69 ± 15.12		
ABC294640	IC_{50}	37.00 ± 7.42	48.08 ± 3.68	32.99 ± 0.92	34.65 ± 0.35
	LC_{50}	>100	>100	76.52 ± 0.20	79.40 ± 4.88

Supplementary Table S3. Antiproliferative activity of single PLX4032 and ABC294630 and their combination in HT-29r cells. Data are expressed as means ± standard deviation from three independent biological experiments.

		ABC294640		
		**35** μM	**17.5** μM	**8.75** μM
**PLX4032**	IC_{50} (μM)	6.19 ± 1.89	16.64 ± 1.68	19.33 ± 1.49
	LC_{50} (μM)	36.40 ± 1.58	>17.5	>8.75

Supplementary Table S4. Combination index (CI) values <1 indicate synergy; CI=1 represents additivity and CI >1 indicates antagonistic effects of combined treatment in HT-29r cells.

HT-29r		
Opaganib (μM)	PLX4032 (μM)	**CI**
8.75	2.5	**0.61**
8.75	5	**0.78**
8.75	10	**0.77**
8.75	20	**0.82**
17.5	2.5	**0.87**
17.5	5	1.03
17.5	10	**0.99**
17.5	20	**0.99**
**35**	2.5	1.19
**35**	5	1.21
**35**	10	1.14
**35**	20	**0.87**

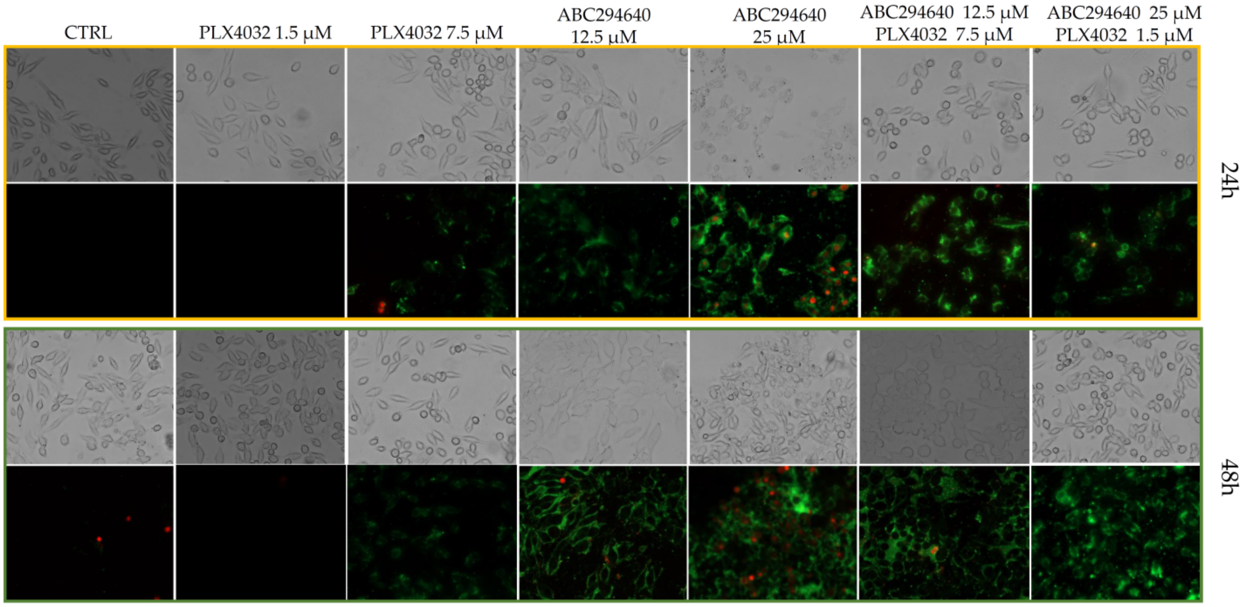

Supplementary Figure S4. Representative microscopic images of apoptosis detection in cells treated with single or combination treatment of vemurafenib (PLX4032) and ABC294640 in RKOr cells using Annexin V-FITC/PI staining. Upper quadrants show light microscopic images of the cells at 10x magnification, while lower quadrants show the cells viewed under a fluorescence microscope at the 10x magnification. Early apoptotic cells (Annexin V+/PI−) are stained green, late apoptotic cells (Annexin V+/PI+) are green and red, and necrotic cells (Annexin V−/PI+) are coloured red.

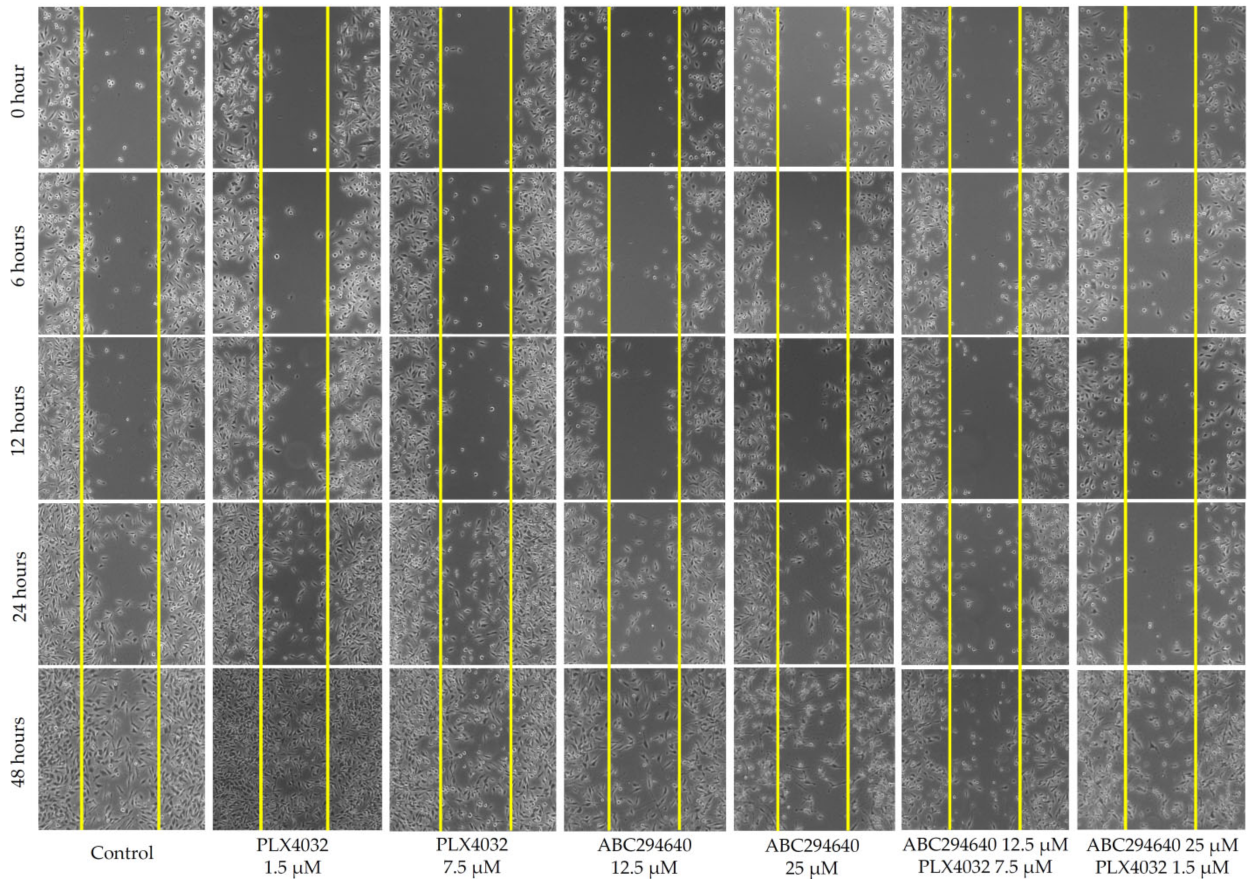

Supplementary Figure S5. Representative light microscopy images captured at 5x magnification, showing the wound closure process indicative of cell migration at different time points in RKOr cells treated with either single agents or combination of PLX4032 and ABC294640.

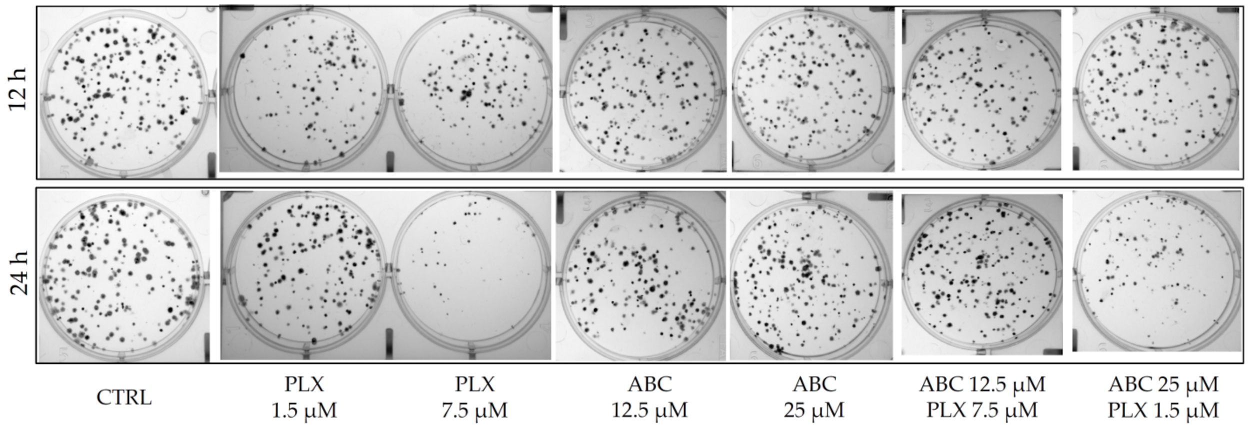

Supplementary Figure S6. Representative images of RKOr cells colonies formed after single or combination treatment with PLX4032 and ABC294640 for 12 and 24 hours. CTRL (Untreated cells), PLX (PLX4032), ABC (ABC294640).
